# Adult Psychotic Symptoms, Their Associated Risk Factors and Changes in Prevalence in Men and Women Over a Decade in a Poor Rural District of Kenya

**DOI:** 10.3390/ijerph120505310

**Published:** 2015-05-19

**Authors:** Rachel Jenkins, Caleb Othieno, Linnet Ongeri, Bernards Ogutu, Peter Sifuna, James Kingora, David Kiima, Michael Ongecha, Raymond Omollo

**Affiliations:** 1 Health Services and Population Research Department, Institute of Psychiatry, Kings College London, de Crespigny Park, London SE 5 8AF, UK; 2 Department of Psychiatry, University of Nairobi, P.O. Box 19676-00202, Kenya; E-Mail: cjothieno@uonbi.ac.ke; 3 Kenya Medical Research Institute, Nairobi, P.O. Box 54840-00200, Kenya; E-Mails: longeri@kemri.org (L.O.); ogutu6@gmail.com (B.O.); romollov@yahoo.co.uk (R.O.); 4 Kombewa Health and Demographic Surveillance Systems, Kombewa, P.O Box 54-40100, Kisumu, Kenya; E-Mail: psifuna@yahoo.com; 5 Kenya Medical Training College, Nairobi, P.O. Box 30195, GPO-00100, Kenya; E-Mail: jkmboroki@yahoo.com; 6 Kenya Medical Research Institute, Kisian, Kisumu P.O. Box 1578-40100, Kenya; E-Mail: michaelongecha@yahoo.com; 7 Ministry of Health, Nairobi P.O. Box 30016, GPO-00100, Kenya; E-Mail: dmkiima@gmail.com

**Keywords:** psychotic symptoms, prevalence, risk factors, repeat survey, time trend, Kenya

## Abstract

There have been no repeat surveys of psychotic symptoms in Kenya or indeed subSaharan Africa. A mental health epidemiological survey was therefore conducted in a demographic surveillance site of a Kenyan household population in 2013 to test the hypothesis that the prevalence of psychotic symptoms would be similar to that found in an earlier sample drawn from the same sample frame in 2004, using the same overall methodology and instruments. This 2013 study found that the prevalence of one or more psychotic symptoms was 13.9% with one or more symptoms and 3.8% with two or more symptoms, while the 2004 study had found that the prevalence of single psychotic symptoms in rural Kenya was 8% of the adult population, but only 0.6% had two symptoms and none had three or more psychotic symptoms. This change was accounted for by a striking increase in psychotic symptoms in women (17.8% in 2013 compared with 6.9% in 2004, *p* < 0.001), whereas there was no significant change in men (10.6% in 2013 compared with 9.4% in 2004, *p* = 0.582). Potential reasons for this increase in rate of psychotic symptoms in women are explored.

## 1. Introduction

Psychotic disorders are prevalent across the world and, although less frequent than common mental disorders, they often have higher severity, disability, chronicity and premature death from suicide and physical illness, so social and economic costs are high [1]. As large samples are required for conditions of relatively low prevalence, there are few epidemiological household studies of psychosis in poorer regions, particularly Africa [2]. Where surveys of psychosis have been conducted, the prevalence rates found are broadly similar to those in the developed world [2].

Three main methods have been used to conduct surveys of psychosis: firstly using clinician administered instruments [3] which can establish both psychotic symptom severity and diagnostic category; secondly using family reports [4]; and thirdly using systematic assessment of psychotic symptoms by detailed interviews administered by non-medical interviewers, leading to enumeration of symptom frequency and severity, and estimates of probable psychosis [5,6,7].

Repeat epidemiological surveys using different samples drawn from the same sample frame at different time points are useful to establish trends in prevalence over time. This paper reports the prevalence of psychotic symptoms and their risk factors in a survey of a new sample drawn in 2013 from the same sample frame as that used for the first epidemiological household study of psychotic symptoms in the same area of Kenya a decade earlier in 2004 [7].

## 2. Materials and Methods

The study design was a community study of the prevalence of psychotic symptoms, and their risk factors in the general population in Nyanza province, near Lake Victoria in Kenya. The study was part of a wider project to examine the associations of malaria, mental disorders and lowered immunity in the adult population. The sample was drawn from the same sample frame as an earlier study in 2004, enabling a comparison of prevalence rates and sociodemographic risk factors.

### 2.1. Study Population

The sample frame is a subdistrict in Kenya, in an area endemic for malaria, namely Maseno area within Kisumu County, Nyanza Province, Western Kenya which has a population of 70,805 [8]. Females constitute 53% of the population. The mean household number is four people per household with a population density of 374 people/km^2^. The population is largely young with a mean age of 23 years. Just under half of the population (46%) are aged under 15, while the other half (49%) are aged 15–64, and the remaining 5% are aged 65 and over.

The population is primarily black African, and the languages spoken are Luo (predominant ethnic group), Kiswahili and English. The area is largely rural, with most residents living in villages, which are a loose conglomeration of family compounds near a garden plot and grazing land. The majority of the houses are mud-walled with either grass thatched or corrugated iron-sheet roofs. Water is sourced mainly from community wells, local streams and the lake for those living on the shores of Lake Victoria. Most water sources are not chlorinated. Subsistence farming, animal husbandry and fishing are the main economic activities in the area. Malaria is holoendemic in this area, and transmission occurs throughout the year. The “long rainy season” from late March to May produces intense transmission from April to August. The “short rainy season” from October to December produces another, somewhat less intense, transmission season from November to January.

### 2.2. Study Site

**Figure 1:**
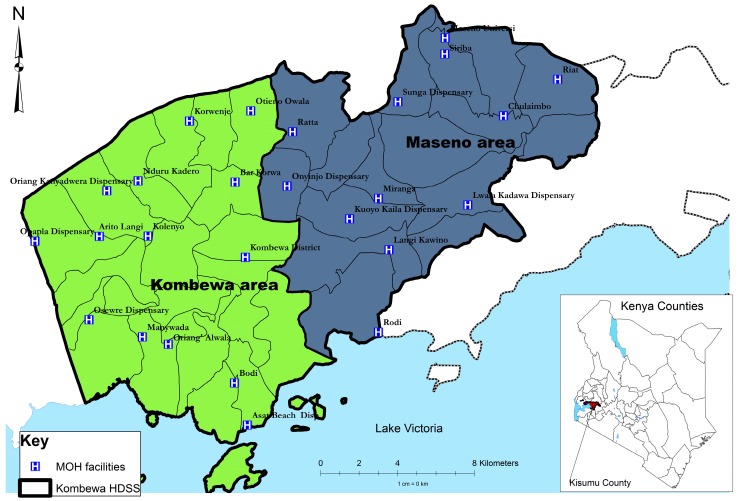
Location of the study site.

The study site is shown in Figure 1:

### 2.3. Study Participants

The study sample was selected from Maseno Area within Kisumu County, western Kenya. Maseno Area is sub-divided into four locations, 17 sub-locations and 184 enumeration areas (villages) based on mapping work done earlier by the Kombewa Health and Demographic Surveillance System (Kombewa HDSS) run by the KEMRI/Walter Reed Project. The Kombewa HDSS is a longitudinal population registration system set up to monitor the evolving health and demographic problems of the study population in Kombewa and Maseno areas [8]. Some villages with less than 50 households were merged together to create new enumeration areas. A random sample of seven households was drawn from each enumeration area, to give a projected sample of 1190 households, which with an estimated response rate of 85% would give a total sample size of 1010. Village maps were used to assign households and guide the research assistants during the survey. Using the Kish Grid Method [9], one individual was selected from each of the sampled household. The demographics and reasons for the refusal were recorded in notebooks by the Research assistants. A total of 1190 households were visited.

### 2.4. Study Procedures

Meetings were held with community leaders to explain the purpose of the survey, and answer questions. The heads of the sampled households, and then the identified participants in the survey were approached for informed consent to the interview. The interview was administered by one of a group of 20 research assistants using a PDA, on which the interview questions were programmed in English, Kiswahili and Luo, and interview responses were recorded. The research assistants received a 5 day training course. During the training a common understanding was developed and adapted of some of the technical words and phrases not commonly used locally. The research assistants were supervised daily in the field by a field manager.

The participants received a structured epidemiological assessment of common mental disorders, psychotic symptoms, alcohol and substance abuse, accompanied by additional sections on socio-demographic data, life events, social networks, social supports, disability/activities of daily living, quality of life, use of health services, and service use, adapted from the questions used in the UK adult psychiatric morbidity survey programme [10].

Demographic information collected included age, sex, ethnicity, marital status and household status (head, spouse or other). Socio-economic factors assessed included employment status, education attainment, economic assets and type of housing.

Common mental disorders were assessed by the Clinical Interview Schedule- Revised (CIS-R) which is a gold standard instrument for use by lay interviewers to assess psychopathology in community settings [11]. It has been widely used in both high [12,13,14] and low income countries [15,16,17], including neighbouring Tanzania [18,19] as well as Kenya [20]. The CIS-R measures the presence of 14 symptom-types in the preceding month and if present, the frequency, duration and severity of each symptom in the past week. Scores taken together with algorithms based on the ICD-10 [21], provide diagnoses of depressive episode (mild, moderate or severe), obsessive compulsive disorder, panic disorder, phobic disorder, generalised anxiety disorder and mixed anxiety/depressive disorder. For the purpose of the current paper however, a score of 12 or more across the 14 sections of the survey was considered an indication of “any CMD”, as used in other CIS-R studies [10].

Psychotic symptoms were assessed with the Psychosis Screening Questionnaire (PSQ) [22], which has also been used previously in Tanzania [23] and Kenya [7]. The PSQ assessed the past year presence of psychotic symptoms. The instrument developed for use by lay interviewers employs five probes to determine recent experience of mania, thought insertion, paranoia, strange experiences and hallucinations. Questions were asked about current and life time use of alcohol, tobacco and drugs. The Alcohol Use Disorders Identification Test (AUDIT) [24] measured hazardous alcohol use.

Respondents were given a list of 11 different stressful life events and asked to say which, if any, they had experienced in the last six months. The list included health risks (serious illness, injury or assault to self or close relative), loss of a loved one (death of a relative; death of a close friend), relationship difficulties (separation or divorce; serious problem with a close friend or relative); income instability (being made redundant or sacked; having looked for work for over a month; loss of the equivalent of three months income) and legal problems (problems with the police involving a court experience; something of value lost or stolen). The list was developed for the British psychiatric morbidity survey programme [12,25], and slightly modified for the east African context [23]. Scores were grouped into either “none”, “one”, “two” and “three or more” life events and were also analysed by category.

Perceived lack of social support was assessed from respondents’ answers to seven questions which were used in the 1992 Health Survey for England [26], and the ONS Surveys of Psychiatric Morbidity [12,25]. The seven questions take the form of statements that individuals could say were not true, partly true or certainly true for them in response to the question “There are people I know who”: (i) Do things to make me happy; (ii) Who make me feel loved; (iii) Who can be relied on no matter what happens; (iv) Who would see that I am taken care of if I needed to be; (v) Who accept me just as I am; (vi) Who make me feel an important part of their lives; and (vii) Who give me support and encouragement. Results were categorised into no, moderate or severe lack of perceived social support.

Social network size was assessed by respondents answers to three questions which have also been used in the ONS surveys of psychiatric morbidity, namely: (i) How many adults who live with you do you feel close to; (ii) how many relatives aged 16 or over who do not live with you do you feel close to; (iii) how many friends or acquaintances who do not live with you would you describe as close or good friends. Responses were added into a total social network score.

Specific questions were also asked about caring responsibilities (Do you give care due to long term physical or mental disorder or disability? And if yes, time spent giving care in a week.); about growing up with one natural parent or two until age 16; and about spending time in an institution before the age of 16.

The instruments used in the 2013 survey were identical to that of the 2004 survey, except that the specific questions above about caring responsibilities, growing up with natural parents, and spending time in an institution were not included in the 2004 survey. The 2004 survey was conducted using pencil and paper interviews while the 2013 survey was conducted using a PDA.

### 2.5. Statistical Analysis

We examined the prevalence of individual non-psychotic symptoms, and their predictors using STATA [27] to produce unadjusted and adjusted odds ratios. Households have been categorized into different socio-economic levels using an index of household assets, constructed applying the principal component analysis procedure, as a proxy indicator for socio-economic status. In developing the asset quintiles, type of house, roofing & walling material, source of water, toilet facility and land have been used [28,29]. The questions on life events and social networks were included in the 2004 survey but provided so much missing data that they could not be analysed, and so these variables were included in the analysis of risk factors in 2013 but not in 2004.

### 2.6. Ethics

Ethical approval was granted by the KCL and KEMRI boards of research ethics respectively (PNM/11/12-54, SSC2374), and permission was obtained to conduct the study in households in Maseno area, which is part of the KEMRI/WRP Kombewa HDSS. Written and witnessed informed consent was asked of participants to take part in the study, and of heads of households for participants’ data to be connected to household data collected by Kombewa HDSS.

## 3. Results

1190 households were selected, and 1158 participants consented to the study while 32 refused to participate in the study interviews, giving a response rate of 97.3%. The prevalence of individual psychotic symptoms and of total PSQ score is given in Table 1.

**Table 1:** Prevalence of individual psychotic symptoms and of total psychotic symptom scores, measured by the PSQ.

Psychosis Screening Questionnaire Items		N (%)
Felt very happy indeed without a break for days on end:n(%)		64 (5.7)
Ever felt like your thoughts are interfered with or controlled: n (%)		14 (1.3)
Ever felt that people are against you: n (%)		75 (6.7)
Ever felt that something strange was going on: n (%)		35 (3.1)
Heard or seen things that others couldn’t: n (%)		21 (1.9)
Total PSQ score	0	965 (86.1)
	1 and above	156 (13.9)
	2 and above	43 (3.8)
	3 and above	8 (0.7)
	4	2 (0.2)

The prevalence of at least one psychotic symptom was 13.9% with only 3.8% having at least two symptoms. Table 2 shows the relationship of various risk factors with the presence of one or more psychotic symptoms. Psychotic symptoms were more common in women (OR 1.8, *p* = 0.001), single people (OR1.8, *p* = 0.009), those with recent life events (OR 2.3 *p* < 0.001 for 2–3 life events in the last 6 months and OR 2.1 *p* = 0.003 for 4 or more life events in the last 6 months), in those with any CMD (OR 3.7, *p* < 0.001), and in those who were not living with both natural parents up to the age of 16 (OR 1.5, *p* = 0.057), while rates were reduced (at borderline level of significance) in older age groups especially those over 60 (OR 0.6, p = 0.056) and in those with low perceived social support (OR 0.08, *p* = 0.04).

**Table 2:** Prevalence of psychotic symptoms and relationship of presence of psychotic symptoms (PSQ 1+) with socio-demographic economic and social variables (social supports, social networks and life events) using bivariate analysis (unadjusted odds ratios).

Factors		N	Prevalence of Psychotic Symptoms (%)	Unadjusted OR (95% CI)	*p*-Value
One or more psychotic symptom *		1121	156 (13.9)		
Sex	Male	595	63 (10.6)	1	-
	Female	524	93 (17.8)	1.8 (1.29 to 2.57)	0.001
Age group	<30 years (yrs)	274	48 (17.5)	1	-
	30–60 yrs	442	59 (13.4)	0.7 (0.47 to 1.10)	0.129
	>60 yrs	167	18 (10.8)	0.6 (0.32 to 1.06)	0.056
Household size	≤6 people	563	73 (13.0)	1	-
	>6 people	556	83 (14.9)	1.2 (0.84 to 1.65)	0.344
Marital Status	Married/cohabiting	698	86 (12.3)	1	-
	Single	180	36 (20.0)	1.8 (1.16 to 2.73)	0.009
	Widowed/divorced	240	34 (14.2)	1.2 (0.77 to 1.80)	0.461
Education	None	121	18 (14.9)	1	-
	Primary	612	78 (12.8)	0.8 (0.48 to 1.45)	0.526
	Secondary	318	45 (14.2)	0.9 (0.52 to 1.70)	0.846
	Post secondary	68	15 (22.1)	1.6 (0.76 to 3.47)	0.214
Employment status	Unemployed	545	80 (14.7)	1	-
	Self employed	477	65 (13.6)	0.9 (0.64 to 1.31)	0.631
	Employed	97	11 (11.3)	0.7 (0.38 to 1.45)	0.387
Asset Groups	Lowest, Q1	397	59 (15.3)	1	-
	Q2	395	50 (12.7)	0.8 (0.54 to 1.21)	0.303
	Highest, Q3	329	47 (13.7)	0.9 (0.58 to 1.34)	0.556
Perceived social support	No lack : 0	3	2 (66.7)	1	-
	Moderate lack: 1–7	309	43 (13.9)	0.08 (0.07 to 0.91)	0.042
	Severe lack: 8+	804	111 (13.8)	0.08 (0.07 to 0.89)	0.040
Size of primary support group	0–3	140	16 (11.4)	1	-
	4–8	506	67 (13.2)	1.2 (0.66 to 2.11)	0.571
	9 or more	470	73 (15.5)	1.4 (0.80 to 2.54)	0.229
Number of life events in last 6 months	0–1	345	28 (8.1)	1	-
	2–3	466	80 (17.2)	2.3 (1.49 to 3.70)	<0.001
	4+	308	48 (15.6)	2.1 (1.28 to 3.43)	0.003
Any CMD	No	1007	119 (11.8)	1	-
	Yes	112	37 (33.0)	3.7 (2.38 to 5.70)	<0.001
Current alcohol	No	1016	139 (13.7)	1	-
	Yes	103	17 (16.5)	1.2 (0.72 to 2.16)	0.431
Hazardous drinking	No	1048	144 (13.7)	1	-
	Yes	71	12 (16.9)	1.3 (0.67 to 2.43)	0.458
	Positive	262	39 (14.9)	1.1 (0.74 to 1.67)	0.601
Carer for more than 4 hours a week	No	26	2 (7.7)	1	-
	Yes	168	34 (20.2)	3.0 (0.69 to 13.5)	0.143
Spent time in institution before age 16	No	895	118 (13.2)	1	-
	Yes	217	36 (16.6)	1.3 (0.87 to 2.0)	0.194
Did not have both natural parents at home until age 16	No	945	123 (13.0)	1	-
	Yes	167	31 (18.6)	1.5 (0.99 to 2.35)	0.057

* Psychotic symptoms as measured by the five domains of the Psychosis Screening Questionnaire (PSQ).

Variables significant at the bivariate level were fed into an adjusted analysis, which is shown in Table 3.

**Table 3:** Final predictive model of psychotic symptoms using logistic regression analysis with odds ratios adjusted for all variables significant in the bivaraiate analysis.

Variable		Adjusted OR (95% CI)	*p*-Value
Sex (=female)		1.7 (1.18 to 2.53)	0.005
Marital Status	Single	2.0 (1.28 to 3.13)	0.002
	Widowed/divorced	0.6 (0.40 to 1.05)	0.078
Number of life events	2–3	2.6 (1.61 to 4.13)	<0.001
	4+	1.9 (1.15 to 3.19)	0.013
Perceived social support	Moderate lack of support	0.1 (0.01 to 1.25)	0.075
	Severe lack of support	0.1 (0.01 to 1.46)	0.097
Any CMD		4.0 (2.45 to 6.56)	<0.001

In the final model, being female (OR 1.7, *p* = 0.005), single (OR 2.0, *p* = 0.002) , having recent life events (OR 2.6, *p* < 0.001 for 2–3 life events and OR 1.9, *p* = 0.013 for four or more life events), and having any CMD (OR 4.0, *p* < 0.001) were significantly associated with presence of one or more psychotic symptoms, while perceived lack of social support and not having both natural parents at home before age 16 were no longer significant.

Table 4 shows that overall prevalence of one or more psychotic symptoms changed from 8.1% in 2004 to 13.9% in 2013 (*p* = 0.001) and that while there was no significant change in male prevalence, the female prevalence changed from 6.9% in 2004 to 17.8% in 2013 (*p* < 0.001).

**Table 4:** Changes in prevalence of presence of psychotic symptoms (PSQ 1+) in men and women between 2004 and 2013.

Psychotic Symptoms	Sex	Prevalence: 2004	Prevalence: 2013	*p*-Value *
PSQ (1+)	Total	8.1	13.9	0.001
	Male	9.4	10.6	0.582
	Female	6.9	17.8	<0.001

* *p*-value from test on equality of proportions.

Table 5 shows that the individual life events associated with an increased risk of having psychotic symptoms (*p* < 0.05) are Serious illness, injury or assault to a close relative (OR 1.6, *p* = 0.006); Major financial crisis, like losing an equivalent of 3 months income (OR 1.7, *p* = 0.006); Bullying (OR 2.1, *p* = 0.025); Violence at home (OR 1.6, *p* = 0.015) and Running away from your home (OR 3.4, *p* = 0.004) while Serious illness, injury or assault to self was associated with a somewhat reduced risk (OR 0.7, *p* = 0.043).

Further analysis (not shown here) of life events by sex on the risk of having psychotic symptoms, found that there were four individual life events that were associated with increased risk of having psychotic symptoms in females, namely serious illness, injury or assault to self, bullying, violence at home and running away from your home while none of the same or other life events were associated with increased risk of having psychotic symptoms in males.

**Table 5:** Risk of Psychotic Symptoms by Life Events.

Factors		N	Prevalence: n (%)	Unadjusted OR (95% CI)	*p*-value
Prevalence of PTSD		1121	156 (13.9)		
Serious illness, injury or assault to self	No	791	121 (15.3)	1	-
	Yes	328	35 (10.7)	0.7 (0.44 to 0.99)	0.043
Serious illness, injury or assault to a close relative	No	794	96 (12.1)	1	-
	Yes	325	60 (18.5)	1.6 (1.16 to 2.34)	0.006
Death of an immediate family member of yours	No	431	58 (13.5)	1	-
	Yes	688	98 (14.2)	1.1 (0.75 to 1.52)	0.711
Death of a close family friend or other relative	No	669	101 (15.1)	1	-
	Yes	450	55 (12.2)	0.8 (0.55 to 1.11)	0.174
Separation due to marital differences, divorce or steady relationship broken	No	1088	151 (13.9)	1	-
	Yes	31	5 (16.1)	1.2 (0.45 to 3.16)	0.722
Serious problem with a close friend, neighbour or relative	No	1021	141 (13.8)	1	-
	Yes	98	15 (15.3)	1.1 (0.63 to 2.01)	0.683
Being made redundant or sacked from your job	No	1066	152 (14.3)	1	-
	Yes	53	4 (7.6)	0.5 (0.17 to 1.38)	0.177
Looking for work without success for >1 month	No	972	134 (13.8)	1	-
	Yes	147	22 (15.0)	1.1 (0.68 to 1.79)	0.700
Major financial crisis, like losing an equivalent of 3months income	No	858	106 (12.4)	1	-
	Yes	261	50 (19.2)	1.7 (1.16 to 2.43)	0.006
Problem with police involving court appearance	No	1082	152 (14.1)	1	-
	Yes	37	4 (10.8)	0.7 (0.26 to 2.12)	0.578
Something you valued being lost or stolen	No	963	133 (13.8)	1	-
	Yes	156	23 (14.7)	1.1 (0.67 to 1.74)	0.755
Bullying	No	1066	143 (13.4)	1	-
	Yes	53	13 (24.5)	2.1 (1.10 to 4.02)	0.025
Violence at work	No	1071	150 (14.0)	1	-
	Yes	48	6 (12.5)	0.9 (0.37 to 2.10)	0.768
Violence at home	No	911	116 (12.7)	1	-
	Yes	208	40 (19.2)	1.6 (1.10 to 2.42)	0.015
Sexual abuse	No	1113	156 (14.0)	1	-
	Yes	6	0 (-)	-	-
Being expelled from school	No	1086	150 (13.8)	1	-
	Yes	33	6 (18.2)	1.4 (0.56 to 3.41)	0.477
Running away from your home	No	1093	147 (13.5)	1	-
	Yes	26	9 (34.6)	3.4 (1.49 to 7.76)	0.004
Being homeless	No	1105	154 (13.9)	1	-
	Yes	14	2 (14.3)	1.0 (0.23 to 4.64)	0.970

## 4. Discussion

This repeat mental health epidemiological survey of a household population in Maseno district, Nyanza province in Kenya, using the same sampling methodology and instruments, has found that the prevalence of one or more psychotic symptoms in this district has risen over the last decade and is relatively high at 13.9% with one or more symptoms and 3.8% with two or more symptoms, with significantly higher rates in women (OR 1.7, *p* = 0.005), single people (OR 2.0, *p* = 0.002) , those with recent life events (OR 2.6, *p* < 0.001 for 2–3 life events and OR 1.9, *p* = 0.013 for four or more life events), and those with CMD (OR 4.0, *p* < 0.001) after adjustment for other sociodemographic variables, substance abuse and malaria.

In a relatively stable high income country such as the UK , no such increase in psychotic symptoms has been found in the series of British national mental health surveys in 1993, 2000 and 2007 [30]. It is difficult to find other countries that have repeated national surveys of psychotic symptoms which might indicate time trends, but comparison with recent surveys shows considerable variability between countries. Singapore conducted a relatively recent mental health survey and found that the life time prevalence was 3.8% [31] while Finland found life time rates of 3.8%, both using a combination of screening questionnaire and case register data [32]. Catalonia found rates of 11.85 [33] and Australia found that 11.7% had at least one psychosis screening symptom [34]. South Africa found a prevalence of 12.7% hallucinations, with no association with age, gender or marital status [35]. There is also variability between ethnic groups within the same country, such as the UK [36].

These 2013 findings of 13.9% having one or more psychotic symptoms, 3.8% having two or more and 0.7% having three or more symptoms represents a significant overall increase in morbidity from the first epidemiological study of psychosis in Kenya [7] which found that the prevalence of single psychotic symptoms in rural Kenya was 8% of the adult population, but only 0.6% had two symptoms and none had three or more psychotic symptoms in this sample size. In contrast, three successive surveys in the UK have found no change in prevalence of psychotic symptoms [30].

Two other studies have also found surprisingly high rates of psychotic symptoms in young people in Kenya in recent years [37,38]. Persecutory ideation and hallucinations has been found elsewhere to be associated with victimisation (bullying, violence and sexual assault) [5,39,40]. In the 2004 Kenya survey, psychotic symptoms were significantly associated with presence of common mental disorders, and to a lesser extent with poor physical health and housing type [7], whereas in this 2013 survey, psychotic symptoms were related to being female, single and having two or more life events (the Kenya 2004 survey was unable to report on life events due to too much missing data in that part of the survey.)

In this 2013 survey, we found a striking increase in psychotic symptoms in women since 2004 (17.8% in 2013 compared with 6.9% in 2004, *p* < 0.001) which is comparable to the increase in CMD in women over the same decade (17.5% in 2013 compared with 10.8% in 2004). We found no significant change in psychotic symptoms in men (10.6% in 2013 from 9.4% in 2004, *p* = 0.582), compared to a fall in CMD in men (paper submitted) (3.8% in 2013 from 10.9% in 2004) [20]. The reasons for this significant increase in risk of psychotic symptoms in women deserve further exploration and research.

We found a relationship of psychotic symptoms with life events. It is possible that this apparent increase in prevalence of psychotic symptoms is related to increasing violence due to political unrest [41,42], and poverty [43]. We found that the individual life events associated with a significantly increased risk of having psychotic symptoms are Serious illness, injury or assault to a close relative, Major financial crisis, like losing an equivalent of 3 months income, Bullying, Violence at home, and Running away from home. When we analysed life events by sex on the risk of having psychotic symptoms, there were 4 individual life events that were associated with increased risk of having psychotic symptoms in females, namely serious illness, injury or assault to self, bullying, violence at home and running away from your home while none of the same or other life events were associated with increased risk of having psychotic symptoms in males. Our data therefore suggest that violence related events and poverty have more effect on risk of psychotic symptoms in women than in men. Unfortunately we were not able to compare these associations with the 2004 situation as there was too much missing data on life events in 2004 to be satisfactorily analysed and reported.

Increased risk of psychotic disorder has long been associated with adverse life events and social disadvantage. In urban Tanzania [23], individuals experiencing two or more stressful life events in the past year had increased risk of psychotic symptoms.

Physical abuse in childhood among British women made them twice as likely as controls to report psychotic symptoms (with lesser effect for sexual abuse) [44]. Exposure to trauma in South Africa [45] and exposure to trauma with intention to harm (e.g., bullying) as a child led to an increased incidence of psychosis in Britain [46].

Another possible reason is concern about the HIV epidemic, which also has a major impact on women. HIV risks are especially severe for young women, where among 15–19-year-olds, females are nearly four times more likely to be infected than males (2.7% to 0.7%) [47]. We unfortunately did not include a question in the survey about HIV concern. Social isolation as well the burden of care giving to the HIV infected and the orphans may also be an explanation, but our data did not show a significant relationship with social networks, perceived social support or caregiving.

Early sexual debut is related to increased rates of HIV infection in young women but not in young men. Women who reported in 2008–2009 that they were less than 16 when they had their first sexual intercourse were more than twice as likely to be HIV-positive as women who began having sex at a later age [47]. Young women in Nyanza Province are especially prone to early sexual debut, with an average age of first intercourse of 16.5 years, compared to 20.3 years for women in Nairobi [47].

A recent national survey of the 2007/8 election related violence in Kenya [42] found 50% of households reporting at least one episode of physical or sexual violence, with women affected more than men, and that all forms of sexual violence increased during the election period. Compared to pre-election, election related sexual violence incidents/1000 persons/year increased over 60 fold and opportunistic sexual violence increased 37-fold. The study report does not disaggregate its data by region, but Nyanza is known to have experienced some of the worst election violence in the country.

It has been suggested that it is culturally more acceptable for women to be expressive about their difficulties while men are expected to bear their problems with greater self control and to be reluctant to admit symptoms of distress. However, it has been demonstrated that the higher rate of symptoms reported by women reflect actual differences in symptoms and not the greater willingness of women to discuss their problems with others [48,49,50]. Indeed controlling for three forms of response bias (naysaying, perceived trait desirability and need for social approval) actually increased the difference between the sexes [50]. Furthermore there is no reason to suppose that women would be relatively more likely than men to yea say in 2013 than in 2004.

Culture can affect what is identified as a hallucination, hallucinations are often culturally meaningful, hallucinations occur at different rates in different settings; and culture affects the meaning and characteristics of hallucinations associated with psychosis [51]. Furthermore it has been suggested that Euro-American culture reduces the reported rate of hallucinations because the shared culture strives to clarify and distinguish whether a given experience is real or imaginary, whereas many non-Western societies do not make such a rigid distinction between reality and fantasy. One might expect, then, that hallucinations would be more readily reported outside of the Western setting. Indeed, the prevalence of hallucinations in the general population varied significantly across different UK ethnic groups [36].

On the other hand while psychotic symptoms may be manifestations of culturally acceptable reactions to trauma exposure, dissociation, and anxiety [52], they may still be clinically significant, and that there is no conclusive evidence to suggest that all heightened incidence rates are solely a function of widespread misdiagnosis. A recent meta-analysis found increased risks of schizophrenia incidence in nearly every migrant group analyzed across a variety of migrant groups and settings, which suggests that social context likely plays a role in the variability of psychotic disorder across populations [53].

We were not able to find any other specific epidemiological studies of psychotic symptoms among the Luo, but a recent commentary indicates that among the Luo communicating with ancestors and hearing their voices is accepted as normal [54]. They believe that a human being is made up of both invisible (shadow) and visible parts (body). When a person dies the visible part disintegrates whereas the invisible part becomes the spirit. The living dead play an important part in the daily lives of their relatives and may communicate their displeasure and haunt the living especially when they are not treated respectfully [54]. Thus communications from these sources in the form of voices or even visual hallucinations may be accepted as normal in the Luo cultural context. The last two items of the PSQ ask whether the person has noticed something strange going on, and whether the person has heard or seen things that others couldn’t. In the cultural situation where hearing voices from the ancestors is regarded as normal, then the respondent who is in fact hearing voices may not answer yes to the question about hearing or seeing things that others couldn’t. If this is the case then the screening questions may in fact underestimate the occurrence such psychotic features [55]. It has also been noted by anthropologists that hallucinations may suddenly increase in a social group at a particular time [55].

Psychosis is more prevalent in the perinatal period [56], but this would not account for the increase in psychotic symptoms in women but not in men between 2004 and 2013 as there has been no corresponding increase in birth rate [57].

Whatever the causes of the increased rates of psychotic symptoms, there is a clear need for further monitoring and for assessment and management at the levels of primary care and district clinics. Some progress has already been made to provide effective continuing professional development on mental health for primary care and district health staff which reached around 50% of primary health care staff between 2005–2011, but this needs to be further rolled out and sustained [58,59,60,61]. Since women are more at risk for psychotic symptoms in this population, an important step would be to include mental health screening at antenatal and postnatal visits.

The strengths of the study include the use of a health and demographic surveillance site for the random sample of households, the high response rate, and the systematic approach to the sociodemographic and clinical assessments, including screening of psychotic symptoms. The population in the surveillance site is regularly monitored by Kombewa HDSS field staff who visit each household bi-annually to capture health and demographic information (Birth rates, Death rates, Causes of Death, Pregnancies, Immunization status, in-and out-migrations *etc*.). Various studies nested on the DSS platform take advantage of the sampling frame inherent in the HDSS, whether at individual, household/compound or regional levels. This familiarity of households with survey procedures is likely to have been influential in the achievement of a high response rate to a relatively lengthy interview procedure but in any event, response rates in this population tend to be high and the previous 2004 survey, undertaken before the establishment of the DSS platform, achieved a response rate of 87.6%. It was probably also helpful that the project team leaders met for the day with the local community leaders to discuss the rationale and procedures for the study a month before the study started.

The survey appraises the presence of psychotic symptoms using the PSQ, but for a definitive diagnosis of psychosis an interview by a trained clinician using an instrument such as the SCAN [62] would be additionally required. The PSQ was not originally designed for Sub-Saharan Africa, and so, as for the previous 2004 survey, it was therefore carefully scrutinised by local clinicians for content validity within the local cultural context, but has not yet been tested in Kenya against a gold standard interview. Similarly, the other components of the adult psychiatric morbidity survey schedule have not been separately assessed for local validity by comparison with gold standard clinical research assessments. However all the scales used in this study and their individual items have been reviewed by local clinicians in Kenya and Tanzania in 2004 [7,19,20,23] and again in Kenya in 2013 for this study, and considered to have content validity. As always, the potential for measurement error when using screening instruments should be acknowledged, given self-reported experiences may be subject to recall or social desirability or cultural response bias [63].

Implementation of the study was hampered by a number of logistical challenges which included the difficult terrain, posing problems for local transport for research staff, especially during rainy periods; and continuing administrative difficulties, which led to delays in the implementation of the project. The interviewing period, initially planned to last 3 months, took place over a period of 6 months, and was temporarily halted for several weeks over the period of the 2013 election due to further fears of election unrest. Finally, the current findings are specific to Maseno division in Nyanza Province and are not necessarily applicable to other parts of Kenya, particularly urban areas.

## 5. Conclusions

This repeat mental health epidemiological survey of a household population in Maseno district, Nyanza province in Kenya has found that the prevalence of one or more psychotic symptoms in this district has risen over the last decade and is relatively high at 13.9% with one or more symptoms and 3.8% with two or more symptoms. We found significantly higher rates in women (OR 1.7, *p* = 0.005), single people (OR 2.0, *p* = 0.002), those with recent life events (OR 2.6, *p* < 0.001 for 2–3 life events and OR 1.9, *p* = 0.013 for four or more life events), and those with CMD (OR 4.0, *p* < 0.001) after adjustment for other sociodemographic variables and substance abuse.

The increase in prevalence since 2004 is significantly greater in women than men, and the reasons for this gender related increase in risk deserve further exploration and research.

Whatever the causes of the increased rates of psychotic symptoms, there is a clear need for further monitoring and for assessment and management at the levels of primary care and district clinics. Some progress has already been made to provide continuing professional development on mental health for primary care and district health staff, but this needs to be further rolled out and sustained. Since women are more at risk for psychotic symptoms in this population, an important additional step is to include mental health screening at antenatal and postnatal visits.
